# Empirical estimation of resource constraints for use in model-based economic evaluation: an example of TB services in South Africa

**DOI:** 10.1186/s12962-018-0113-z

**Published:** 2018-07-30

**Authors:** Fiammetta M. Bozzani, Don Mudzengi, Tom Sumner, Gabriela B. Gomez, Piotr Hippner, Vicky Cardenas, Salome Charalambous, Richard White, Anna Vassall

**Affiliations:** 1 Department of Global Health and Development, London School of Hygiene & Tropical Medicine, 15-17 Tavistock Place, London, WC1H 9SH UK; 2 The Aurum Institute, Johannesburg, South Africa; 3 TB Modelling Group, TB Centre, CMMID, London School of Hygiene & Tropical Medicine, London, UK; 4 Department of Infectious Disease Epidemiology, London School of Hygiene & Tropical Medicine, London, UK

**Keywords:** Constraints, Economic evaluation, Mathematical modelling, Health systems, Tuberculosis, South Africa

## Abstract

**Background:**

Evidence on the relative costs and effects of interventions that do not consider ‘real-world’ constraints on implementation may be misleading. However, in many low- and middle-income countries, time and data scarcity mean that incorporating health system constraints in priority setting can be challenging.

**Methods:**

We developed a ‘proof of concept’ method to empirically estimate health system constraints for inclusion in model-based economic evaluations, using intensified case-finding strategies (ICF) for tuberculosis (TB) in South Africa as an example. As part of a strategic planning process, we quantified the resources (fiscal and human) needed to scale up different ICF strategies (cough triage and WHO symptom screening). We identified and characterised three constraints through discussions with local stakeholders: (1) financial constraint: potential maximum increase in public TB financing available for new TB interventions; (2) human resource constraint: maximum current and future capacity among public sector nurses that could be dedicated to TB services; and (3) diagnostic supplies constraint: maximum ratio of Xpert MTB/RIF tests to TB notifications. We assessed the impact of these constraints on the costs of different ICF strategies.

**Results:**

It would not be possible to reach the target coverage of ICF (as defined by policy makers) without addressing financial, human resource and diagnostic supplies constraints. The costs of addressing human resource constraints is substantial, increasing total TB programme costs during the period 2016–2035 by between 7% and 37% compared to assuming the expansion of ICF is unconstrained, depending on the ICF strategy chosen.

**Conclusions:**

Failure to include the costs of relaxing constraints may provide misleading estimates of costs, and therefore cost-effectiveness. In turn, these could impact the local relevance and credibility of analyses, thereby increasing the risk of sub-optimal investments.

**Electronic supplementary material:**

The online version of this article (10.1186/s12962-018-0113-z) contains supplementary material, which is available to authorized users.

## Background

Frameworks for priority setting for the control of infectious diseases in low-and middle-income countries are evolving. In recent years, mathematical models of disease transmission have been increasingly used for supporting priority setting efforts in these settings [1–3]. At the same time, the importance of considering both supply-side and demand-side constraints on the uptake, delivery and cost-effectiveness of global health interventions has been widely recognised [4, 5]. Understanding constraints is particularly relevant in low and middle-income countries, where non-financial constraints such as human resources scarcity may substantially impact the feasibility of implementation and pace of scale-up of interventions [6, 7]. Traditionally, model-based priority setting has incorporated and adapted to local demographic and epidemiological characteristics, but to date the explicit consideration of the impact of context-specific health system constraints on the costs and cost-effectiveness of global health interventions is often absent [8].

Several approaches have been proposed for incorporating both financial and non-financial resource constraints in model-based priority setting for infectious diseases. These allow the analyst to either restrict outputs to limit the impact of interventions, or to cost the relaxation of constraints. For limiting impact, some have adopted an ‘integrated modelling’ approach combining disease and operational (health systems) modelling. However, this approach has substantial data requirements as detailed knowledge of numerous processes across the health system is necessary to populate the operational model [9, 10]. Another approach is mathematical programming, which examines solutions that maximise global health objectives under a range of constraints [11]. Mathematical programming has the advantage of potentially dealing simultaneously with multiple constraints, such as equity and efficiency [12–14]. However, combining this approach with infectious disease models is computationally complex and data-intensive [14]. While mathematical programming has the potential to be widely applied in the presence of strengthened health information systems, the ‘black box’ nature of this approach may constitute a barrier for users and result in a process that lacks transparency for decision-makers [15].

A gap remains for approaches to support decision-makers set priorities that assess the impact of constraints and that are feasible within planning timeframes and under considerable data scarcity. In this context, we present a proof of concept for a pragmatic method for the empirical estimation of both financial and non-financial constraints from routine data. Target users are analysts involved in priority setting to support national strategic planning processes, who wish to explicitly model the impact of selected constraints. This method was developed and piloted during the policy planning process for the 2017–2022 National Tuberculosis Plan (NTP) in South Africa, rather than in a research setting. We illustrate the approach in respect of facility-based tuberculosis (TB) case-finding strategies. Specifically, we present methods for estimating financial, human resource and diagnostic constraints, and demonstrate how these can be used in estimating the costs of the facility-based TB case finding strategies under constraints.

## Methods

### Study setting

South Africa faces one of the world’s worst TB epidemics [16]. TB programmes often rely on passive case-finding, focused on the screening of individuals presenting to health facilities with TB symptoms. This method has been shown to miss a large proportion of facility-based TB cases presenting with unrelated symptoms [17]. In 2015, Intensified Case Finding (ICF) for TB was adopted in South Africa for detecting TB cases among HIV-infected health facility attendees, who are screened for TB at each facility visit. During the previous year, the South African TB Think Tank was established with the purpose of advising the National Department of Health (NDoH) on TB policy and strategic planning [18]. As part of this effort, an economic analysis to identify the optimal ICF and TB diagnostic strategy for South Africa was conducted. The ICF programme would be targeting over 100 million visits to primary health care facilities per year.

### Identifying and characterising constraints

**Fig. 1:**
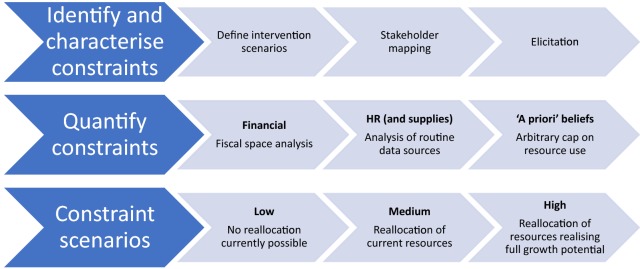
Summary of constraints estimation process

The analysis concentrated on supply-rather than demand-side constraints. The process of constraints estimation is described in Fig. 1. Three constraints on health system resources relevant to TB service provision were identified through discussions with local stakeholders that primarily took place as part of the TB Think Tank meetings (NDoH TB programme managers and staff, technical assistants and local TB experts supporting the national planning processes). Stakeholders were selected based on their participation in the TB Think Tank. Individual discussions were held with 12 Think Tank members and group discussions were facilitated during Think Tank meetings, attended by an average of 25 stakeholders. In the context of policy meetings, no formal interview process was used. In addition to financial constraints, stakeholders consistently highlighted human resource and supplies constraints and agreed for these to be considered in the analysis.

### Financial constraint

We conducted a fiscal space analysis for estimating the financial constraint for TB. Baseline levels of total public expenditure on TB were estimated using the most recent figures (2013) [19]. To convert South African Rand into US Dollars, we used the average official exchange rate over the 3-year period to 2016 (13.9) [20]. For the NTP 2017–2022 period, we used the fiscal space model developed by Remme et al. [21]. This model considers the increase in public spending from GDP growth, health prioritisation within the overall government budget as well as TB prioritisation within overall health spending, earmarked alcohol taxes and efficiency gains.

While in principle resources for TB could be allocated from the general health budget, the budgeting process is historically incremental. We therefore considered three scenarios (Table 1), including one where policy makers do not use all the fiscal space available to them. In the low constraint scenario (most limiting), the TB budget was assumed to grow at an annual rate of 1.7% in line with GDP growth only [21]. This assumes that TB policy makers are unable to advocate for a greater share of the overall health budget. In the medium constraint scenario, we assumed that, between 2017 and 2022, an increased share (from 2 to 15%) of the health budget is allocated to TB to match the share of the burden of disease caused by TB, adjusted for TB mortality as reported by the NDoH (15%). In the high constraint scenario (least limiting), we assumed that 15% of a health budget that achieves its full fiscal space growth (3%) is allocated to TB. In all scenarios we assumed that, post 2022, TB expenditure would continue to grow by a constant rate of 1.7% per annum.

**Table 1:** Projected annual growth in budget and human resources for TB under different constraint scenarios, from most to least limiting

Year	Budget constraint			Human resource constraint		
	Low (%)	Medium (%)	High (%)	Low (%)	Medium (%)	High (%)
2015	–	–	–	–	–	–
2016	1.7	1.7	1.7	0.9	0.9	0.9
2017	1.7	47.5	51.8	0.8	4.2	4.2
2018	1.7	32.2	34.1	0.8	4.1	4.1
2019	1.7	24.4	25.4	0.7	3.9	3.9
2020	1.7	19.6	20.3	0.7	3.7	3.7
2021	1.7	16.4	16.9	0.7	3.6	3.6
2022	1.7	1.7	1.7	0.6	0.6	3.1
2023	1.7	1.7	1.7	0.6	0.6	3.1
2024	1.7	1.7	1.7	0.6	0.6	3.1
2025	1.7	1.7	1.7	0.6	0.6	3.2
2026	1.7	1.7	1.7	0.5	0.5	3.2
2027	1.7	1.7	1.7	0.5	0.5	3.2
2028	1.7	1.7	1.7	0.5	0.5	3.2
2029	1.7	1.7	1.7	0.5	0.5	3.2
2030	1.7	1.7	1.7	0.5	0.5	3.3
2031	1.7	1.7	1.7	0.5	0.5	3.3
2032	1.7	1.7	1.7	0.5	0.5	3.3
2033	1.7	1.7	1.7	0.5	0.5	3.3
2034	1.7	1.7	1.7	0.5	0.5	3.3
2035	1.7	1.7	1.7	0.5	0.5	3.4

### Human resource constraint

In consultation with the NDoH, the constraint on human resources was characterised as the available annual full time equivalent (FTE) of nursing staff to provide all TB services, expressed in minutes of available nursing time. The constraint was applied to all TB services, as increased screening will also increase the use of other TB services along the patient pathway. Nurses are the main cadre of staff that deliver TB case detection, diagnosis and treatment in South Africa. The nursing cadres considered were professional (or registered) nurses, including TB and antiretroviral therapy nurses; and enrolled nurses, who are not specialised and carry out many of the same activities as registered nurses but do not prescribe drugs. The analysis was limited to nurse availability in primary health care (PHC) clinics and health centres.

The total FTE spent by nursing staff on TB in 2015 (baseline) was estimated from routine data available upon request (DHIS, Persal, Electronic TB Register). In addition, estimates were informed by ongoing or recently concluded costing studies that measured human resource use, published literature and personal communications with the NDoH (see Additional file 1 for further details). Projections on the growth of the nursing workforce in South Africa were informed by historical data available from the South African Nursing Council [22].

Three human resource constraint scenarios were considered (Table 1). For the low (most limiting) constraint, we assumed TB policy-makers are unable to re-allocate nurses from other services. In this case, the maximum annual minutes spent on TB were held at the current level and adjusted in future years based on population growth projections, holding the ratio of nurses trained per capita constant [23]. For the medium and high constraints, nurse time was allocated to TB according to the disease burden (from 6 to 15%) during the NTP period. This was calculated as the ratio of total deaths from TB among HIV-infected and -uninfected patients [24], to total deaths in South Africa [25]. Post 2022, the low and medium constraint increased annually in line with population growth projections. The high (least limiting) constraint also took the historically rapid nursing workforce growth in South Africa into account.

### Diagnostic supplies constraint

Strengthening TB case-finding may have a substantial impact on diagnostic supplies requirements and related costs. While South Africa has demonstrated the capacity to rapidly scale up TB diagnostic volumes [26], in consultation with the NDoH we identified a constraint on the amount of TB diagnostic supplies (Xpert MTB/RIF tests) purchased annually. This constraint corresponds to the rule of thumb that has been used to set the TB diagnostic budget in previous years (South African NDoH, personal communication), which limits the TB programme to a ratio of 20 Xpert MTB/RIF tests for every case of TB diagnosed.

### Estimating the costs of TB services considering constraints

**Fig. 2:**
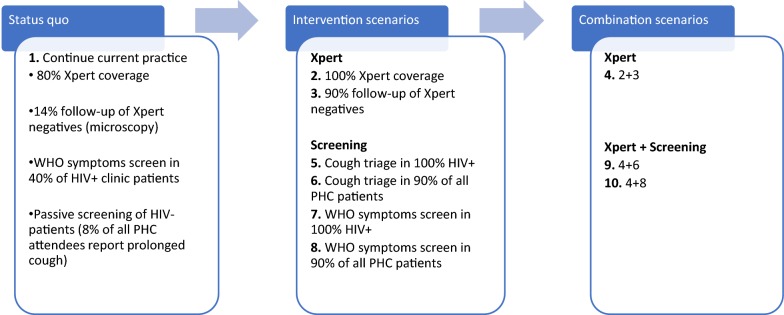
Modelled interventions

We estimated TB service costs both under constraints and with relaxing of the constraints using a mathematical model of TB that estimates TB cases, TB mortality and the use of TB services [27]. The model was calibrated for the year 2015 and cost projections were generated for a 20-year period up to 2035. The model was used to calculate costs of introducing nine different ICF intervention options (described in Fig. 2), by multiplying projected TB service volumes by the unit costs for all TB services and interventions.

In detail, the ICF intervention options examined involve screening two different populations (all clinic patients or only those that are HIV-infected) using either cough triage, a single question on whether the respondent has been coughing for at least 2 weeks, or the full WHO symptoms screener, whereby patients reporting either current cough, fever, weight loss or night sweats are referred for TB testing [28]. Unit costs for TB services and interventions in South Africa were derived from previously published data or constructed using ingredients costing, as described in Additional file 1. A discount rate of 3% was applied to future costs.

The model was first used to estimate costs of the ICF interventions in an unconstrained environment, expanding services to coverage levels defined by policy targets (as described elsewhere and in Additional file 1) [27]. Each constraint scenario was then applied independently. When constraints were exceeded, the model was re-run at a reduced coverage, such that the projected costs or (in the case of the human resource constraint) nurse time remained below the constraint over the entire time horizon. For the supplies constraint, coverage was limited once the ratio of diagnostic tests to TB notifications was exceeded. Results on the effects of the constraints on intervention impact and the resulting coverage gaps are described elsewhere by Sumner et al. [27].

Relaxing the Xpert MTB/RIF constraint was assumed to have no costs aside from purchasing and deploying additional tests. Similarly, relaxing the financial constraints results in no additional costs compared to the unconstrained scenario. The additional costs of relaxing the human resource constraint was estimated as the cost of hiring and employing the extra nurses needed to supply the additional number of minutes required for the three constrained scenarios to reach the coverage and output levels achieved under the unconstrained scenario. Our estimations considered a mix of registered (51%) and enrolled (49%) nurses based on DHIS and South African Nursing Council data. In discussion with the NDoH, some of the extra minutes required to relax the constraints were supplied by existing private sector nurses, as the average monthly salary for public sector nurses is higher than that in the private sector [29]. The underlying assumption is that, currently, nurses join the private sector due to a lack of open positions in the better paying public sector. In the first year where the nurse minutes requirements exceeded availability under the constrained scenario, we assumed all minutes worked in primary health care by private sector nurses could be re-allocated to the public sector as needed. In subsequent years, we assumed the historical proportion of new graduates joining the private sector would join the public sector instead. All private sector nurses and nursing graduates were assumed to be registered nurses.

The additional minutes needed to relax the constraints that could not be covered by employing the current private sector workforce were costed by estimating the costs of increasing government-sponsored new graduates. Professional nurses spend a total of 48 months in training while enrolled nurses take 36 months to qualify [30]. Both cadres receive a monthly stipend of US$ 676 from the government to cover living expenses while in training. Once employed, a 10% mark-up on the annual salary of new graduates was factored into take the transaction costs of employment into account. An additional 10% mark-up over basic pay was added for 30% of new nurses, representing the allowance received by those posted in rural areas [31].

## Results

### Base case estimates of financial, human resources and number of Xpert MTB/RIFs

In 2015, we estimate a public expenditure for the TB programme of approximately U$ 415 million, of which US$ 310 million was spent on direct service provision and the rest on administration and other above-service-level activities. Changes in financial resource requirements for service provision over time in the absence of new interventions are shown in Table 2. The decrease in the costs of TB services over time under this scenario reflects the projected declining trend in the TB epidemic in South Africa.

**Table 2:** Base case financial resource requirements for TB services provision, 2016 US$

Year	Passive case finding	ICF—cough triage	ICF—WHO symptoms screener	Xpert negatives follow-up	First line treatment	MDR-TB treatment	Xpert MTB/RIF diagnosis	Smear microscopy diagnosis	IPT	Total
2015	1,410,773	16,735,538	11,339,995	719,731	40,180,219	116,780,734	104,264,301	8,788,407	10,075,340	310,295,039
2016	1,361,274	16,163,576	13,152,808	713,400	38,432,227	116,926,226	110,340,641	9,300,580	12,055,578	318,446,309
2017	1,315,734	15,417,893	17,142,665	726,632	37,705,880	118,058,283	125,981,140	10,618,913	16,398,177	343,365,316
2018	1,275,370	14,936,907	18,332,255	712,543	35,898,981	114,934,319	129,548,686	10,919,620	17,722,633	344,281,313
2019	1,240,571	14,555,234	18,524,339	694,551	34,356,526	111,743,996	129,002,723	10,873,601	17,967,440	338,958,982
2020	1,209,293	14,214,061	18,368,678	676,625	33,071,298	109,171,196	127,087,494	10,712,167	17,835,516	332,346,329
2021	1,180,102	13,891,486	18,096,024	659,396	31,946,882	107,155,267	124,747,372	10,514,919	17,576,279	325,767,726
2022	1,152,180	13,578,567	17,787,797	642,850	30,920,793	105,540,398	122,303,518	10,308,927	17,277,749	319,512,780
2023	1,125,078	13,271,337	17,471,075	626,861	29,958,551	104,197,245	119,855,790	10,102,609	16,969,529	313,578,075
2024	1,098,620	12,968,654	17,155,575	611,357	29,044,267	103,043,788	117,439,480	9,898,939	16,662,200	307,922,880
2025	1,072,710	12,669,940	16,844,627	596,290	28,169,833	102,027,241	115,065,298	9,698,820	16,359,422	302,504,180
2026	1,047,337	12,375,476	16,540,194	581,648	27,331,230	101,115,987	112,741,285	9,502,929	16,063,288	297,299,373
2027	1,022,536	12,086,063	16,242,380	567,427	26,526,100	100,291,602	110,469,170	9,311,413	15,773,812	292,290,502
2028	998,299	11,801,897	15,950,333	553,607	25,752,333	99,540,603	108,244,943	9,123,934	15,490,056	287,456,007
2029	974,636	11,523,309	15,663,396	540,179	25,008,440	98,855,029	106,066,036	8,940,275	15,211,297	282,782,598
2030	951,549	11,250,556	15,380,642	527,129	24,292,808	98,228,624	103,928,488	8,760,101	14,936,554	278,256,451
2031	929,014	10,983,533	15,101,333	514,434	23,603,600	97,655,673	101,828,214	8,583,070	14,665,078	273,863,947
2032	907,022	10,722,349	14,824,025	502,070	22,938,847	97,130,242	99,758,485	8,408,613	14,395,348	269,587,000
2033	885,559	10,466,925	14,548,635	490,027	22,297,387	96,649,025	97,718,406	8,236,655	14,127,320	265,419,939
2034	864,608	10,217,086	14,275,791	478,295	21,678,167	96,209,490	95,710,021	8,067,369	13,861,714	261,362,541
2035	844,149	9,972,617	14,006,160	466,869	21,080,136	95,809,377	93,735,448	7,900,933	13,599,291	257,414,979

*ICF* intensified case-finding, *MDR-TB* multi-drug resistant tuberculosis, *IPT* isoniazid preventive therapy

**Fig. 3:**
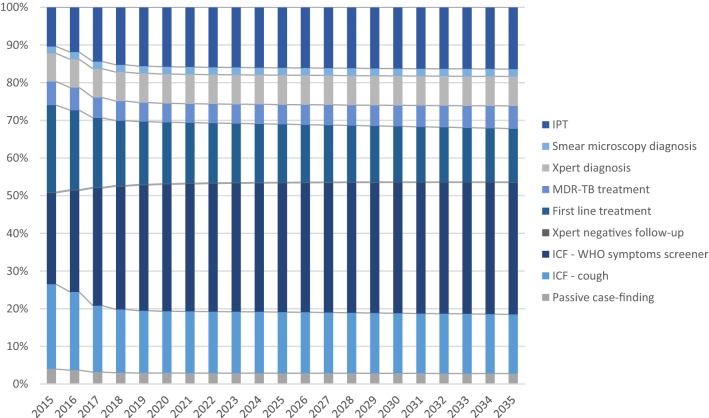
Base case human resource time for TB services provision, share of total minutes

We estimate that the South African nursing workforce supplied approximately 209.5 million minutes to TB services in public primary health care facilities in 2015. Specialised TB nurses accounted for about 15% of total minutes for TB, while most TB service provision time was supplied by other professional nurses (51%) and enrolled nurses (34%). Figure 3 shows the distribution of staff time across different TB services under the base case, assuming no new intervention is introduced. Finally, the number of Xpert MTB/RIF tests in 2015 was estimated to be 3.2 million.

### Constrained scenarios

**Fig. 4:**
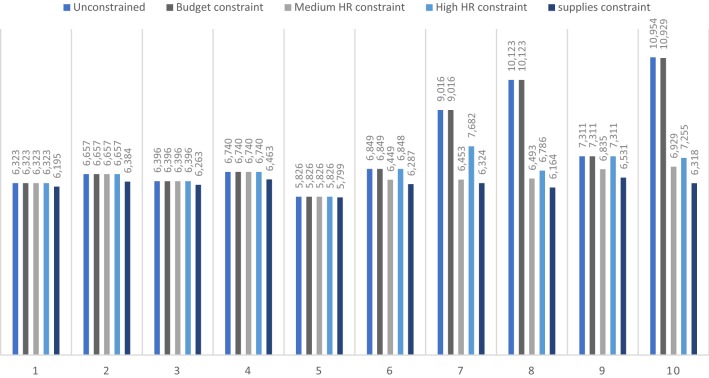
2016–2035 budget requirements for interventions at reduced coverage under financial, human resource and Xpert constraints, 2016 US$ (millions). Interventions are described in Fig. 2

The low (most limiting) budget and human resource constraints were exceeded by almost all interventions. These constraints were deemed unrealistic to overcome and thus not modelled further, as they made any expansion of case-finding infeasible without substantial disinvestment in other areas of TB care. Conversely, the high budget constraint was never exceeded. The impact of the other constrained scenarios on TB costs between 2016 and 2035 is presented in Fig. 4. Under the medium budget constraint, all case-finding interventions could be ‘afforded’ with the exception of intervention 10 (combination of expanding Xpert MTB/RIF coverage, increasing adherence to the Xpert-negative algorithm and achieving 90% coverage of the WHO symptoms screener among all PHC clinic attendees). Under this scenario, there was a relatively small funding gap of approximately US$ 24.9 million over the period 2016–2035 for intervention 10 (Fig. 4).

The human resource constraints do not impact interventions 2–5 (expanded Xpert MTB/RIF utilisation and increased adherence to the Xpert-negative algorithm, alone or in combination, and cough-based screening among all HIV-infected PHC clinic attendees, respectively). Interventions 6–8 (cough-based screening, both on its own among all PHC attendees and in combination with the strengthening of diagnostic algorithms) are impacted to a limited extent. Implementation of interventions 9 and 10 (combinations of strengthened diagnostic algorithms and ICF using cough triage or the WHO symptoms screener, respectively) is substantially constrained by human resource availability. If this constraint is not addressed, there is a difference in predicted TB costs of more than US$ 4 billion between the unconstrained and medium human resource constraint scenarios over the period 2016–2035 due to the restrictions on the level of TB services that the health system can supply.

The diagnostic supplies constraint restricts the coverage of all interventions, and similarly results in a reduction in costs by limiting access to TB services (Fig. 4).

### Costs of relaxing the human resource constraints

The incremental nurse minutes that would be needed to relax the human resource constraints and match the intervention coverage achieved in the unconstrained scenario are presented in Table 3. We found that a maximum of 3.3 million nurse minutes, corresponding to the annual time supplied by approximately 1300 nurses, can be switched from the private to the government sector between 2017, the year in which the National TB Plan case-finding interventions begin rolling out, and 2035. This is sufficient to cover the incremental needs of cough-based screening interventions (6 and 9) under all constrained scenarios, but does not meet the extra demand generated from strengthening the use of the WHO symptoms screener. Achieving 90% ICF coverage would require approximately 15 or 25% of all nurses in the South African public health system to work on TB if using cough triage or the WHO tool, respectively.

**Table 3:** Incremental nurse minutes and costs of relaxing the human resource constraints for impacted interventions

Interventions	Incremental nurse minutes needed	Nurse minutes available in private sector	Incremental salary costs	Incremental training costs	Total costs of relaxing constraint
Medium constraint					
6	391,839,546	3,288,019	202,071,898	260,963,844	463,035,742
7	2,092,347,066	3,288,019	1,080,038,659	1,464,587,472	2,544,626,131
8	3,223,758,889	3,288,019	1,664,183,076	2,094,657,776	3,758,840,851
9	427,006,436	3,288,019	220,228,454	284,203,098	504,431,552
10	3,184,770,361	3,288,019	1,644,053,420	2,066,650,290	3,710,703,709
High constraint					
6	273,592	273,592	121,813	183,109	304,922
7	1,043,322,764	3,288,019	538,430,664	730,965,942	1,269,396,606
8	2,951,942,738	3,288,019	1,523,845,239	1,925,170,799	3,449,016,039
9	266,569	266,569	118,686	178,280	296,967
10	2,914,419,377	3,288,019	1,504,472,045	1,898,371,197	3,402,843,241

The highest costs of relaxing the constraint arise under the medium constraint scenario for intervention 8, aimed at strengthening the use of the WHO symptoms screener among all PHC attendees. The costs of hiring and training a sufficient number of nurses in this scenario between 2017 and 2035 are approximately US$ 3.76 billion (US$ 187.9 million per year on average), corresponding to approximately 60% of the entire financial resource requirements for delivering the baseline TB services during the same period (US$ 316.2 million per year on average).

## Discussion

We present a pragmatic approach using routine data and stakeholder opinions, applied in a policy context, that was used to inform decision-makers on the impact of supply-side constraints on ICF for TB in South Africa. We were able to produce estimates of constraints and apply these to TB transmission models to limit attainable service coverage and generate estimates of intervention costs, advancing the work conducted by Hontelez et al. [14], who considered a single, non-empirically defined cost of health systems strengthening. We provide an illustration of the work of Van Baal et al., who recommend that human resource constraints should be considered in economic evaluation to avoid producing biased cost-effectiveness estimates that could mislead decision-makers [15]. In the South African setting we find that, when constraints are considered, substantial additional costs may be incurred to expand TB services, which will impact incremental cost-effectiveness ratios.

Conceptually, the constraints analysed in our approach may be considered as ‘policy’ constraints representing ‘fixity’ in resource use, which is primarily determined by policy choice rather than by the inherent characteristics of the resources. In this sense, our analysis highlighted the costs to TB programme managers of policies around financing and human resource planning. Likewise, our analysis could be used by those planning human resources to help estimate the health impact of those investments. Compared to other modelling approaches used in the literature, our method has relatively limited data requirements and is less computationally intensive than operational modelling [9, 10]. While the results may be less comprehensive and not provide formal optimisation within constraints compared to other methods, they can be used to promote deliberation to redefine the mix of health system strengthening with disease specific activities within intervention strategies.

As a proof of concept conducted in a pragmatic rather than research setting, our analysis has many limitations. Several of these need improvement as the methods are further applied. Firstly, our selection of constraints was informal, both in terms of the identification of stakeholders and of the list of constraints identified. This may underestimate the costs of addressing all constraints. Secondly, we assumed that unit costs and minutes per service remain constant during intervention scale-up and we do not consider the differential impact of constraints at different coverage levels. A third limitation is data quality for some of the routine information systems. For example, DHIS data on the annual hours worked by public sector nurses is not updated every year to reflect staff movements to the private sector, thus potentially overestimating nurse minutes supplied in the government sectors.

Our broad approach (stakeholder identification of constraints, routine data and stakeholder engagement to characterise them, independent application in the model and deliberation) is potentially generalisable to other settings. However, the way it is implemented is likely to be highly dependent on both local data sources and policy processes. In South Africa, formal processes for strategic planning are developing and there is no formal health technology assessment. In other settings, it may be possible to substantially improve both the elicitation and characterisation of constraints. Despite the shortcomings of our ‘proof of concept’ approach, the engagement of stakeholders through the whole process helps ensuring ownership, relevance and usefulness of the analysis. This work was used to inform the National TB Strategy, specifically to refine the staffing of ICF approaches [18, 32]. Moreover, the engagement of stakeholders can lay the foundation for further work to refine and develop the analysis, as iterative decisions are made during intervention scale-up.

## Additional file

**Additional file 1.** Technical appendix.
