# Supplementary material for: Empirical estimation of resource constraints for use in model-based economic evaluation: an example of TB services in South Africa
Source: Cost Eff Resour Alloc. 2018 Jul 30;16:27. doi: 10.1186/s12962-018-0113-z (PMC6065151; doi:10.1186/s12962-018-0113-z)
Supplement: Supplementary file 1 — Additional file 1. Technical appendix. [file 12962_2018_113_MOESM1_ESM.docx]

**TECHNICAL APPENDIX**

**Empirical estimation of resource constraints for use in model-based economic evaluation: an example of TB services in South Africa**

Fiammetta M. Bozzani, Don Mudzengi, Tom Sumner, Gabriela B. Gomez, Piotr Hippner, Vicky Cardenas, Richard White, Anna Vassall

**CONTENTS**

[S1. Human resource constraint estimation 2](#_Toc496798561)

[S.1.1 Nursing workforce in South Africa and growth projections 2](#_Toc496798562)

[S.1.2 Minutes per output of National TB Plan interventions 3](#_Toc496798563)

[S.1.3 Total nurse Full Time Equivalent spent on TB services in South Africa 4](#_Toc496798564)

[S.2. Economic model 7](#_Toc496798565)

# S1. Human resource constraint estimation

## S.1.1 Nursing workforce in South Africa

The number of nurses currently working in primary health care (PHC) and the number of students enrolled in nursing courses in South Africa were obtained from the South African Nursing Council (SANC)^[[1]](#footnote-1)^. The SANC database includes all nurses registered for practice in the country in both public and private sectors. The register might thus include nurses who are currently working abroad and those who are retired or otherwise inactive, as well as those who are active but do not spend time with patients (e.g. SANC employees, nurses working for research institutions and on clinical trials etc.). Following the methods described in a published analysis^[[2]](#footnote-2)^ as well as in the latest strategy on human resources for health adopted by the National Department of Health^[[3]](#footnote-3)^, the assumptions presented in Table S1 were used to derive an estimate of the number of active nurses working in PHC in the public sector.

Table S1. Nursing registration parameter assumptions

| **Parameter** | **Value** | **Description** | **Source** |
| --- | --- | --- | --- |
| Annual attrition rate | -25% | Graduates who do not find postings and out migration | NDOH HRH strategy 2012-2017 |
| Annual returning /immigration rate | +4% | Returning migrants and immigrant workers | Assumption |
| Annual retirement rate | -6% | Nurses leaving workforce | NDOH HRH strategy 2012-2017 |
| Graduates joining private sector | -41% | Corresponds to share of private sector workers | NDOH HRH strategy 2012-2017 |
| Inactive nurses | -18% | Share of nurses on SANC register not in contact with patients | Econex 2010 |

Nursing workforce growth projections for the twenty-year period between 2015 and 2035 were based on the observed rate of increase in employed nurses and nurses-in-training registered with SANC over the period 2006-2015. The average annual growth rate was then adjusted by the projected rate of population growth in South Africa^[[4]](#footnote-4)^. The share of nurses working in PHC was assumed to be 94% of all nurses in the public sector^[[5]](#footnote-5)^. The projected numbers of nurses and workforce growth over the period 2015 to 2035 are shown in Table S2.

Table S2. Public sector nursing workforce growth projections, 2015-2035

| **Year** | **PHC nurses** | | | **Average growth rate** |
| --- | --- | --- | --- | --- |
|  | **Registered** | **Enrolled** | **Auxiliaries** |  |
| 2015 | 51,279 | 27,492 | 28,812 | - |
| 2016 | 51,923 | 29,041 | 31,185 | 5.04% |
| 2017 | 52,619 | 30,697 | 33,587 | 4.91% |
| 2018 | 53,340 | 32,468 | 36,021 | 4.80% |
| 2019 | 54,114 | 34,359 | 38,487 | 4.71% |
| 2020 | 54,934 | 36,378 | 40,987 | 4,63% |
| 2021 | 55,800 | 38,536 | 43,522 | 4,56% |
| 2022 | 56,714 | 40,839 | 46,095 | 4,51% |
| 2023 | 57,677 | 43,298 | 48,706 | 4.46% |
| 2024 | 58,690 | 45,924 | 51,358 | 4.42% |
| 2025 | 59,754 | 48,727 | 54,052 | 4.39% |
| 2026 | 60,872 | 51,728 | 56,789 | 4.36% |
| 2027 | 62,043 | 54,910 | 59,572 | 4.33% |
| 2028 | 63,271 | 58,317 | 62,402 | 4.31% |
| 2029 | 64,555 | 61,951 | 65,280 | 4.29% |
| 2030 | 65,898 | 65,828 | 68,209 | 4.28% |
| 2031 | 67,302 | 69,964 | 71,190 | 4.26% |
| 2032 | 68,767 | 74,374 | 74,225 | 4.25% |
| 2033 | 70,295 | 79,076 | 77,316 | 4.24% |
| 2034 | 71,889 | 84,090 | 80,464 | 4.23% |
| 2035 | 73,551 | 89,437 | 83,672 | 4.22% |

## S.1.2 Minutes per output of National TB Plan interventions

The minutes per day spent by nursing staff on different TB services and the number of patients receiving their services in one day were obtained from unpublished MERGE trial data that were collected for the economic evaluation^[[6]](#footnote-6)^. These are summarised in Table S3.

Table S3. MERGE trial estimates of time spent on TB activities, minutes per day

| **HR cadre** | **TB screening** | **Sputum collection** | **IPT** | **TB treatment*** |
| --- | --- | --- | --- | --- |
| Registered nurse, general | 83 | 18 | 54 | 0 |
| Registered nurse, TB | 4 | 81 | 29 | 133 |
| Enrolled nurse | 24 | 0 | 18 | 0 |

* No difference observed between treatment for naïve and retreatment patients

The MERGE data likely underestimate the number of minutes that nursing staff spend on TB activities as these data rely on self-report, and a tendency to over-report the denominator (number of patients seen per day) was observed during data collection. Conversely, the self-report data do not account for multi-tasking, thus potentially leading to an overestimate, since an activity’s duration is shorter when performed simultaneously with another activity.

The interventions defined as part of the National TB Plan modelling exercise were assigned a time per output based on their different components. The minutes per output for each TB service and intervention analysed are reported in Table S4, along with their source and calculation assumptions. Where the time per output of an activity was not available from MERGE, data was integrated with results from the XTEND trial^[[7]](#footnote-7)^ analysis and from a community-based contact-tracing programme in peri-mining communities funded through the Global Fund to Fight AIDS, TB and Malaria, as well as with personal communications with the National Department of Health.

## S.1.3 Total nurse Full Time Equivalent spent on TB services in South Africa

The total minutes worked per day by registered nurses were calculated from the number of working days per year recorded by the District Health Information Software (DHIS). For enrolled nurses, minutes per day were estimated by multiplying the total working minutes by the number of practicing and newly graduated nurses registered with SANC in 2015^[[8]](#footnote-8)^. Parameter assumptions and their sources are summarised in Table S5.

The share of TB nurses out of the total staff at PHC clinics as well as the time spent in contact with TB patients by the different cadres were calculated from MERGE trial data (Table S6).

We then combined this percentage with the total number of minutes worked per day for all nurses to calculate the total annual Full Time Equivalent (FTE) spent on TB. This estimate of TB working time was calibrated to the total staff time spent on TB estimated by the transmission model at baseline, as we were not confident that the MERGE sites were representative of South Africa as a whole. The baseline model estimate was obtained by multiplying model outputs by the number of minutes to deliver a service estimated above (see section A.2.2). We used the time spent on providing TB treatment as the calibration parameter and we assessed the difference between the estimate generated from the MERGE data, calculated based on the observed number of patients per day at the study sites, and the predicted number of patients per year generated by the model.

We estimated that 25% of daily working hours are used as down time, resting and other activities not related to patient care. This was based on the difference in total working time calculated from alternative sources of self-reported data in the MERGE trial (number of TB patients per day and FTE spent on TB, multiplied by the minutes spent on each service and the total expected FTE, respectively).

Table S4. Modelled interventions, minutes per output for professional and enrolled nurses

| **Activities** | **Unit** | **Nurse minutes** | **Source** | **Assumptions** |
| --- | --- | --- | --- | --- |
| Passive case finding | per patient screened | 2.63 | MERGE and XTEND trials | Average of cough question and full symptoms screener duration to account for reported suboptimal screening practices |
| Xpert MTB/Rif diagnosis | per suspect | 3.16 | MERGE trial | Minutes per suspect for sputum collection |
| Smear microscopy diagnosis | per suspect | 3.16 | MERGE trial | Minutes per suspect for sputum collection |
| Follow-up of HIV-infected Xpert negative patients | per HIV+ Xpert negative | 8.61 | MERGE trial | Sum of sputum collection time and duration of two visits, one for monitoring and one for results collection |
| Screening using cough triage | per patient screened | 1.26 | MERGE trial | MERGE uses nurses self-reported data. Screening time per patient, on average lower than in XTEND, was thought to reflect the suboptimal screening practice of cough triage instead of the recommended full WHO symptoms screener. |
| Screening using WHO symptoms screener | per patient screened | 4.00 | XTEND trial | XTEND data are from direct observation, and more likely to reflect the recommended practice. |
| First line TB treatment (initiation phase, 2 months) | per patient month | 35.72 | MERGE and XTEND trial | One monitoring visit with sputum collection and two for drug collection |
| First line TB treatment (continuation phase, 4 months)) | per patient month | 7.57 | MERGE and XTEND trial | Three monitoring visits with sputum collection and two for drug collection |
| MDR-TB treatment, with DOTS (initiation phase, 6 months) *† | per patient month | 237.04 | MERGE and XTEND trial | 25 visit with sputum collection and 132 for drug collection |
| MDR-TB treatment, with DOTS (continuation phase, 18 months) *† | per patient month | 159.83 | MERGE and XTEND trial | 396 visits for drug collection |
| MDR-TB treatment (initiation phase, 6 months) | per patient month | 84.47 | MERGE and XTEND trial | 25 visit with sputum collection for Xpert/microscopy + 31 for drug collection |
| MDR-TB treatment (continuation phase, 18 months) | per patient month | 7.27 | MERGE and XTEND trial | 94 visits for drug collection |
| IPT | per patient month | 5.54 | MERGE trial | One visit per month and one sputum collection per year |

ILTFU: Initial Loss to Follow-Up; IPT: Isoniazid Preventive Therapy

Note: shaded activities represent interventions that are being introduced or modified under the 2017-2022 National TB Plan, as opposed to routine services

* From personal communication from the National Department of Health: 20% of notified TB cases receive treatment under DOTS, others self-medicate and visit the clinic once a month to collect drugs.

† 40% of MDR patients receive decentralised treatment at clinics while the remaining 60% are hospitalised during the intense phase and then go on to receive decentralised care from clinics in the continuation phase. Given that the HR constraint only applies to PHC services, those services provided during the initiation phase on hospitalised MDR-TB patients were omitted from its calculation.

Table S5. Working days parameter assumptions

| **Parameter** | **Description** | **Value** | **Source** |
| --- | --- | --- | --- |
| Annual days worked by registered nurses | FTE provided by PHC nursing staff over 12 months (July 2015 – June 2016 | 3,333,173 | SA DHIS 2016 |
| Annual working days | Allowing for annual leave, public holidays and sickness | 223 | Assumption |
| Daily minutes worked | Full-time work, 8 hours per day | 480 | Assumption |

Table A6. Summary of MERGE trial data on human resources and time use

| **HR cadre** | **Average per PHC facility,**  **N (%)** | **Full time equivalent spent on TB, %** |
| --- | --- | --- |
| Registered nurse, general | 13 (33) | 24% |
| Registered nurse, TB | 1.5 (4) | 70% |
| Enrolled nurse | 2 (4) | 15% |

# S.2. Economic model

The cost model, including all unit costs of routine TB services and of the intensified case finding (ICF) interventions recommended for the 2017-2022 National TB Plan, was developed using data from the published literature as well as from ongoing studies conducted in South Africa. These costs were attached to transmission model outputs to generate estimates of the annual financial resource requirements of the national TB programme.

All costs were converted to 2016 US$ using the South African GDP deflator ^[[9]](#footnote-9)^. Table S7 reports all unit costs used in the economic model, as well as their sources and underlying assumptions.

Table S7. Cost model

| Intervention | Description | Unit | Unit cost of output (2016 US$) | Source |
| --- | --- | --- | --- | --- |
| Nurse time | One minute of professional nurses' time | Per minute | 0.34 | Nicola Foster, unpublished XTEND data |
| Inpatient day | Cost of hospitalisation | Per bed-day | 44.44 | Edina Sinanovic, unpublished Xtend data |
| OPD visit | Nurse consultation, 12 minutes average duration | per event | 4.08 | Nicola Foster, unpublished XTEND data |
| IPT treatment | One OPD visit a month (at half cost as on HIV) + INH + Xpert cost every year | per month | 7.81 | Salome Charalambous, personal communication |
| First line TB treatment | Facility-based observation. 2 months intensive phase, 4 months continuation phase | per patient month | 21.43 | Treatment regimens from The Aurum Institute (2016) ^[[10]](#footnote-10)^. Drug prices from National Department of Health's master procurement catalogue - 8 April 2016 ^[[11]](#footnote-11)^. Only 20% of patients are treated under DOTS, the rest visit facility once a month to collect drugs (Dr Lindiwe Mvusi, National Department Of Health, personal communication). |
| MDR-TB treatment | 6 months intensive phase, 18 months continuation phase | per patient month | 359.06 | As for first line treatment. From Sinanovic et al. (2015) ^[[12]](#footnote-12)^, 40% of patients are hospitalised during intensive phase, the rest receive fully decentralised treatment. |
| TB diagnostics | Sum of costs of first and second line diagnostic tests, including visits and antibiotics * | per person diagnosed | 53.65 | Costs of first line diagnostics from Cunnama et *al.*(2016) ^[[13]](#footnote-13)^. Costs of monitoring tests from Edina Sinanovic, unpublished XTEND data |
| WHO symptoms screener | 4 minutes of a professional nurse | per suspect screened | 1.36 | Nicola Foster, unpublished XTEND data |
| Cough triage | 1.3 minutes of professional nurse asking cough question | per suspect screened | 0.68 | MERGE trial |

OPD: Out-patient Department; IPT: Isoniazid Preventive Therapy; INH: Isoniazid; MDR-TB: Multi-Drug Resistant Tuberculosis

Note: shaded activities represent interventions that are being introduced or modified under the 2017-2022 National TB Plan, as opposed to routine services

* Cost per person diagnosed calculated as a weighted average of the unit costs of each test from the XTEND trial, where the weights represent the probability of receiving each test experienced by diagnosed patients in the XTEND cohort

1. http://www.sanc.co.za/stats.htm [↑](#footnote-ref-1)
2. Econex, (2010). *The Human Resource Supply Constraint: The Case of Nurses* [↑](#footnote-ref-2)
3. NDOH, (2011). HRH strategy for the health sector: 2012/13 - 2016/17 [↑](#footnote-ref-3)
4. http://data.worldbank.org/data-catalog/population-projection-tables [↑](#footnote-ref-4)
5. This is equal to the proportion of annual working days supplied by nurses in PHC over the total, from DHIS data obtained from the National Department of Health upon request [↑](#footnote-ref-5)
6. See Kufa T, Hippner P, Charalambous S, Kielmann K, Vassall A, Churchyard G, Grant AD, Fielding KL: A cluster randomised trial to evaluate the effect of optimising TB/HIV integration on patient level outcomes: the "merge" trial protocol. *Contemp Clin Trials* 2014; 39(2):280-7 [↑](#footnote-ref-6)
7. See Churchyard GJ, Stevens WS, Mametja LD, McCarthy KM, et *al*: Xpert MTB/RIF versus sputum microscopy as the initial diagnostic test for tuberculosis: a cluster-randomised trial embedded in South African roll-out of Xpert MTB/RIF. *Lancet Glob Health* 2015; 3:e450-57 [↑](#footnote-ref-7)
8. http://www.sanc.co.za/stats/Stat2015/Year%202015%20Provincial%20Distribution%20Stats.pdf [↑](#footnote-ref-8)
9. Statistics South Africa. Historical Consumer Price Index. Accessed on 1^st^ May 2017. Available at: http://www.statssa.gov.za/publications/P0141/CPIHistory.pdf? [↑](#footnote-ref-9)
10. The Aurum Institute, (2016). Managing TB in a new era of diagnostics. Available at: <http://www.auruminstitute.org/index.php?option=com_jdownloads&view=categories&Itemid=260>. Accessed on 2 May 2017. [↑](#footnote-ref-10)
11. Available at: <http://www.health.gov.za/index.php/component/phocadownload/category/196>. Accessed September 2016. [↑](#footnote-ref-11)
12. Sinanovic E, Ramma L, Vassall A, Azevedo V, Wilkinson L, Ndjeka N, McCarthy K, Churchyard G, Cox H: Impact of reduced hospitalisation on the cost of treatment for drug-resistant tuberculosis in South Africa. Int J Tuberc Lung Dis 2015, 19(2):172-178 [↑](#footnote-ref-12)
13. Cunnama L, Sinanovic E, Ramma L, Foster N, Berrie L, Stevens W, Molapo S, Marokane P, McCarthy K, Churchyard G et al: Using top-down and bottom-up costing approaches in LMICs: The case for using both to assess the incremental costs of new technologies at scale. Health Econ 2016, 25(S1):53-66 [↑](#footnote-ref-13)
